# Differential and state-dependent effects of the GluN2A-selective positive allosteric modulator GNE-5729 on executive functions

**DOI:** 10.1038/s41386-026-02476-1

**Published:** 2026-06-25

**Authors:** Johannes Lauer, Julia Poppke, Thomas Nickl-Jockschat, Daniela C. Dieterich, Markus Fendt, Samia Afzal

**Affiliations:** 1 Institute for Pharmacology and Toxicology, Otto-von-Guericke University Magdeburg, Magdeburg, Germany; 2 Center of Behavioral Brain Sciences, Otto-von-Guericke University Magdeburg, Magdeburg, Germany; 3 Department of Psychiatry and Psychotherapy, Otto-von-Guericke University Magdeburg, Magdeburg, Germany; 4 German Center for Mental Health (DZPG), Partner Site Halle-Jena-Magdeburg, Magdeburg, Germany; 5 Department of Psychiatry, Iowa Neuroscience Institute, University of Iowa, Iowa City, IA USA

**Keywords:** Ion channels in the nervous system, Cognitive neuroscience

## Abstract

Cognitive deficits in executive functions such as working memory and cognitive flexibility are central to many brain disorders, yet effective treatments remain limited. Dysfunction of GluN2A-containing NMDA receptors (NMDAR) contributes to these impairments, and positive allosteric modulators (PAM) that selectively enhance GluN2A function offer a promising strategy to rescue cognitive deficits while preserving physiological neurotransmission. However, it remains unclear whether PAM-driven GluN2A potentiation can rescue mechanistically distinct, pharmacologically induced impairments and whether the efficacy of GluN2A-PAMs differs across executive domains and sexes. Here, we assessed the effects of GNE-5729, a brain-penetrant GluN2A-selective PAM, on executive functions in mice. GNE-5729 showed no overall effect on working memory performance in the Y-maze, but improved performance in mice with low baseline spontaneous alternations, indicating a baseline-dependent effect. It also rescued working memory deficits induced by dl-amphetamine and scopolamine, which disrupt neuromodulatory regulation while leaving NMDAR channels functionally accessible. In contrast, GNE-5729 failed to reverse MK-801-induced impairments, consistent with MK-801’s non-competitive pore blockade of NMDAR. Notably, GNE‑5729 effects showed sex‑dependent patterns, with female mice displaying more pronounced effects. GNE-5729 had no effects on cognitive flexibility in the attentional set-shifting task (ASST) and did not alter prefrontal GluN2A expression, though it disrupted the association between endogenous GluN2A levels and ASST performance observed in controls. Together, these findings indicate that GNE-5729 exerts effects under conditions of reduced baseline performance or disrupted neuromodulatory signaling and demonstrates differential effects across executive domains, supporting a state-dependent therapeutic profile of GluN2A-targeted modulation.

## Introduction

Executive functions (EF) are higher-order cognitive processes that enable flexible, goal-directed behavior. These functions are critical for everyday decision-making, problem-solving, and self-regulation. Deficits in EF contribute substantially to impairments in daily functioning, underscoring the need for interventions that can restore EF in clinical populations [1, 2]. Among the molecular mechanisms implicated in executive dysfunction, N-methyl-D-aspartate receptors (NMDARs) play a pivotal role due to their critical involvement in synaptic plasticity and cognitive functions [3, 4]. NMDAR dysfunction has been implicated in numerous neuropsychiatric and neurological disorders [5, 6], as well as in normal aging [7], and is associated with cognitive deficits, particularly in core executive domains like working memory [8–10] and cognitive flexibility [11–14].

NMDAR are heterotetrameric complexes typically composed of two obligatory GluN1 and two regulatory GluN2(A-D) subunits [15, 16] with GluN2A becoming the predominant synaptic subunit in the adult forebrain [17–19]. GluN2A-containing receptors are strongly associated with pro-survival signaling and synaptic plasticity [20, 21] making them a notable target for therapeutic modulation. Genetic and pharmacological evidence support a role for GluN2A in EF: GluN2A-knockout mice display deficits in cognitive flexibility [22] and negative allosteric modulation of GluN2A impairs performance in the cognitive flexibility task, including the attentional set-shifting task (ASST) [12]. Moreover, GluN2A expression in the prefrontal cortex (PFC) correlates with ASST performance [12] suggesting that enhancing GluN2A function may represent a viable strategy to rescue cognitive deficits.

Positive allosteric modulators (PAM) that target GluN2A are promising for ameliorating executive dysfunction. GluN2A-selective PAM enhances NMDAR responses only in the presence of the endogenous co-agonists, thereby preserving the spatiotemporal integrity of synaptic transmission and reducing the risk of excitotoxicity. This pharmacological profile makes them particularly attractive for potentially rescuing cognitive impairments. GNE-5729 is a next-generation GluN2A-selective PAM with favourable pharmacokinetics, high brain penetration, and strong subtype selectivity [23] making it a useful tool to investigate the effects of GluN2A-mediated positive allosteric modulation on EF deficits in vivo. Despite its promising pharmacology, it remains unclear whether PAM-driven GluN2A potentiation can improve EF or rescue EF deficits and whether such effects differ across executive domains (e.g., working memory vs cognitive flexibility) or between sexes.

In this study, we investigated the pharmacodynamic effects of GNE-5729 across two executive domains in mice. First, we assessed its dose-dependent effects on working memory in the Y-maze and examined whether individual baseline performance influenced GNE-5729 efficacy. Based on this, we tested whether GNE-5729 could reverse working memory impairments induced by three mechanistically distinct compounds (dl-amphetamine, MK-801, and scopolamine). Second, given evidence linking GluN2A to cognitive flexibility [12, 24], we explored the effects of GNE-5729 on ASST performance following four days of treatment. Finally, we quantified prefrontal GluN2A expression to determine its association with ASST performance under vehicle and GNE-5729 treatment.

## Materials and methods

### Animals and housing conditions

Male and female C57BL/6 J mice were group-housed (≤ 8 per cage, same sex) under controlled conditions (22 ± 2°C; 55 ± 10% humidity; 12 h light/dark cycle, lights on at 6:00 am). Food and water were provided *ad libitum*, except for mice used in the ASST, which were food-restricted (∼2.5 g/mouse/day) starting one week prior to and throughout the ASST to maintain 90–95% of their baseline body weight (bw). All procedures complied with the International Guidelines for the Care and Use of Animals for Experimental Procedures (Directive 2010/63/EU) and were approved by local authorities (Landesverwaltungsamt Sachsen-Anhalt, Az. 42502-2-1618 UniMD). Behavioral experiments were conducted during the light phase (setup brightness: 100-200 lx), beginning between 8:00 and 9:00 am.

### Compound preparation and administration

All compounds were administered at 10 ml/kg bw. GNE-5729 (Hölzel Diagnostika, Cologne, Germany) was dissolved in dimethylsulfoxide (DMSO, Hölzel Diagnostika) and diluted in 20% (2-hydroxypropyl)-β-cyclodextrin (Hölzel Diagnostika) in saline (final DMSO concentration: 10%). For (-)-dizocilpine maleate (MK-801; Hölzel Diagnostika), dl-amphetamine sulphate and scopolamine hydrobromide trihydrate (both from Sigma-Aldrich, Taufkirchen, Germany), saline was used as the vehicle. All solutions were stored at –80 °C until use.

Based on data from a structurally related compound [25], GNE-5729 was tested at doses of 0.75, 1.5, and 3.0 mg/kg. GNE-5729, dl-amphetamine (5 mg/kg) [26] and scopolamine (1 mg/kg) [27] were administered intraperitoneally, whereas MK-801 (0.15 mg/kg) [28] was given subcutaneously. Vehicle solutions served as controls.

### Behavioral experiments

#### Y-maze

The Y-maze test was used to assess working memory in mice [29, 30] by leveraging their innate tendency to explore novel environments. The custom-built apparatus consisted of three identical arms (38 × 8 × 13 cm) positioned 120° apart, with transparent walls, a gray PVC floor, and distinct visual cues to aid spatial orientation. Mice were placed at the end of one arm and allowed 8 min of free exploration without rewards. Arm entries (all four paws inside an arm) were manually recorded, and the maze was cleaned between trials to remove olfactory cues. Spontaneous alternations, defined as three consecutive arm visits in a distinct non-repeating sequence (e.g., ABC, BAC, but not ABA), served as the primary measure of working memory [31, 32]. The percentage of spontaneous alternations was calculated as: (number of spontaneous alternations)/ (total arm entries – 2) ×100. Healthy mice typically exceed the 50% chance level [31, 33] whereas values at or below chance indicate impairment.

#### Attentional set shifting task (ASST)

Cognitive flexibility was assessed using a seven-phase, two-choice digging ASST, as described in detail in the Supplementary Information (section 1.1).

### Experimental procedure

#### Experiment 1: GNE-5729 effects on working memory

To assess the dose-dependent effects of GNE-5729 on working memory, spontaneous alternations were first measured without any pre-treatment. A total of 12 females and 13 males (8–11 weeks) received intraperitoneal injections of GNE-5729 or vehicle. Behavioral testing was conducted 20 min after injection. Each dose was administered in a separate test session in a pseudorandomized order using a Latin square design, with a 3-4 day interval between sessions. Testing was conducted in two sequential experimental runs. In the first run (6 males, 6 females), mice received all three doses in a pseudorandomized order. In the second run (7 males, 6 females), only the 0.75 and 1.5 mg/kg doses and vehicle were used, as the 3 mg/kg dose was excluded due to sedative effects observed in the initial run.

Next, in three independent experiments, mice were pre-treated with either MK-801 (a non-competitive NMDA receptor antagonist), dl-amphetamine (monoamine reuptake inhibitor), or scopolamine (muscarinic acetylcholine receptor antagonist) to induce distinct working memory deficits and evaluate the effects of GNE-5729 modulation on pharmacologically-impaired working memory.

For each experiment, 10 females and 10 males (8-29 weeks) were assigned to four treatment conditions via a crossover design, i.e., vehicle with vehicle, vehicle with a working memory-impairing drug, GNE-5729 with vehicle, and GNE-5729 with a working memory-impairing drug. Treatment order was pseudorandomized using a Latin square design and a 7-day washout period between sessions. Each test session involved two injections: GNE-5729 (1.5 mg/kg) or vehicle administered intraperitoneally 30 min prior to testing, followed by a working memory-impairing drug or its vehicle 20 min before testing.

#### Experiment 2: GNE-5729 effects on cognitive flexibility

12 females and 12 males (10–13 weeks) underwent the ASST. Each mouse received either GNE-5729 (1.5 mg/kg) or vehicle, administered intraperitoneally 20 min before all four daily ASST sessions. Mice completing all phases or failing only a single phase were included in the analysis.

### Molecular analyses

Following the last ASST phase (Rev3), brain dissection, tissue collection, crude synaptosomal membrane preparation, and Western blotting were performed as described in detail in the Supplementary Information (section 1.2).

### Statistical analysis

Data were analyzed using Prism 10 (GraphPad Software Inc., CA, USA) and SYSTAT 13 (Systat Software GmbH, Düsseldorf, Germany). Behavioral data were analyzed using multifactorial repeated-measures ANOVA, followed by Dunnett’s test or the Benjamini, Krieger and Yekutieli procedure. Molecular data were analyzed using non-parametric tests where appropriate. Linear regression was used to assess relationships between baseline performance and GNE-5729 effects, as well as between GluN2A expression and ASST performance. Significance was set at *p* ≤ 0.05. Data are presented as mean ± SEM.

## Results

### GNE-5729 effects on working memory

#### GNE-5729 shows sex-specific, baseline-dependent effects and reduces locomotion at 3 mg/kg

Working memory and locomotion were assessed using the percentage of spontaneous alternations and arm entries, respectively. One outlier was excluded from the analysis of spontaneous alternations (ROUT, Q = 0.2%).

**Fig. 1:**
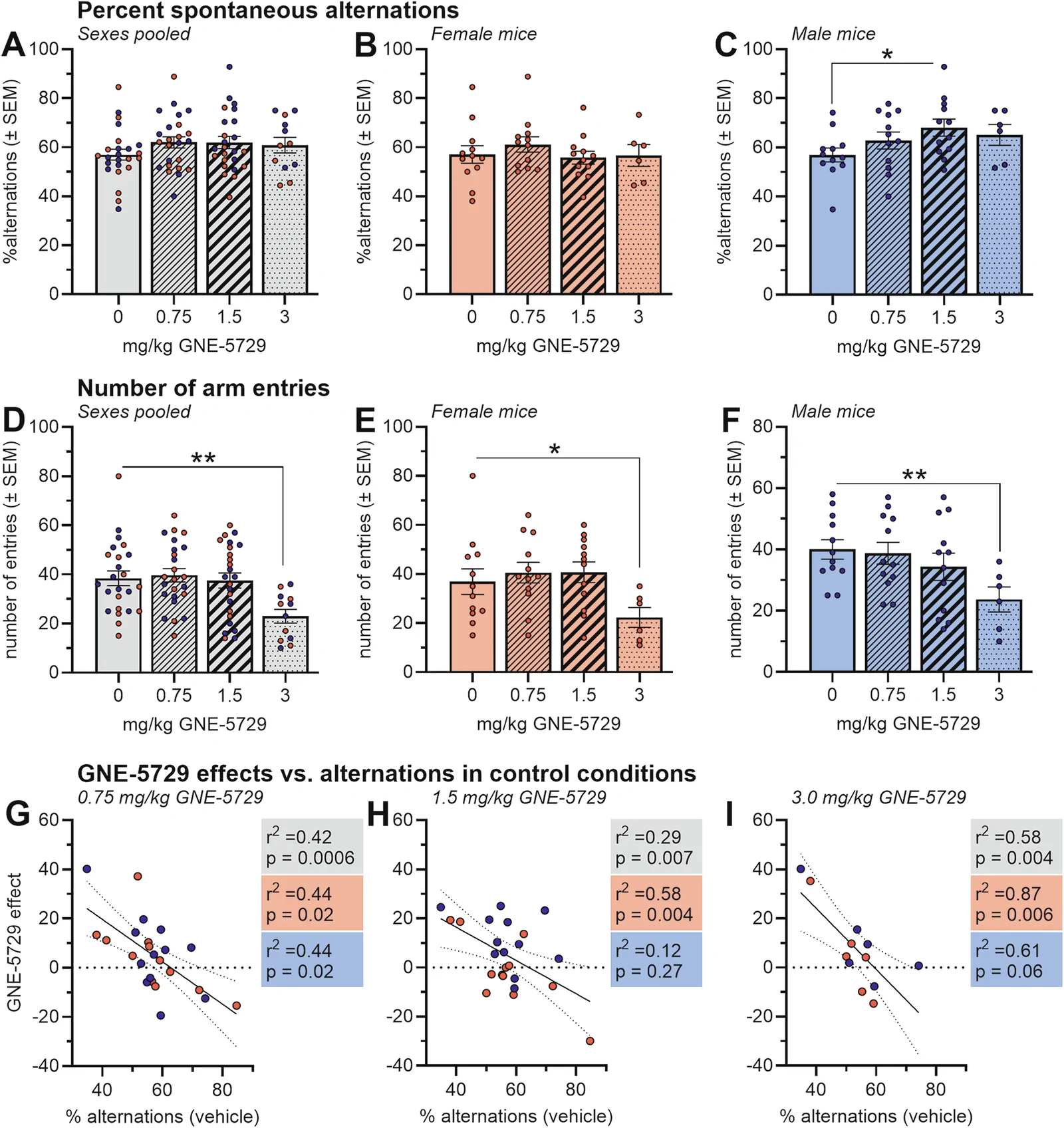
Dose-response assessment of GNE-5729 on spontaneous alternations and locomotor activity. **A**–**C** Percentage of spontaneous alternations following vehicle (0), 0.75, 1.5, and 3.0 mg/kg GNE-5729. No significant treatment effects were observed when females and males were analyzed together (**A**). Separate analyses showed no effects in females (**B**), whereas males exhibited a significant treatment effect with increased spontaneous alternations at 1.5 mg/kg (**C**). **D**–**F** Number of arm entries as a measure of locomotor activity. GNE-5729 significantly reduced locomotion at 3.0 mg/kg when sexes were pooled (**D**), with similar treatment effects observed separately in female (**E**) and male (**F**) mice. **G–I** Baseline-dependent effects of GNE-5729 on spontaneous alternations. All doses showed significant negative correlations, indicating that mice with lower baseline performance exhibited greater improvement following GNE-5729 treatment. Correlation statistics (r², p) for combined (gray), female (red), and male (blue) datasets are shown in the adjacent statistics boxes. * *p* < 0.05, ** *p* < 0.01; post-hoc comparisons following significant ANOVA effects. Data represent mean ± SEM (**A–F**). Individual data points are shown for each mouse (red = female mice, blue = male mice).

The effects of different GNE-5729 doses on spontaneous alternations were analyzed using repeated-measures ANOVA, which showed no main effect of sex (*F*_1,22_ = 3.28, *p* = 0.08, ηp² = 0.13), treatment (*F*_3,54_ = 1.35, *p* = 0.28, ηp² = 0.07), or their interaction (*F*_3,54_ = 1.84, *p* = 0.15, ηp² = 0.09) (Fig. 1A). When analyzed separately, females (Fig. 1B) showed no GNE-5729 effects (*F*_3,27_ = 0.57, *p* = 0.64, ηp² = 0.06), whereas males showed a significant treatment effect (*F*_3,27_ = 2.88, *p* = 0.05, ηp² = 0.24; Fig. 1C). Post-hoc testing revealed that 1.5 mg/kg GNE-5729 significantly increased spontaneous alternations (*q* = 2.85, *p* = 0.02).

Analysis of the number of arm entries using repeated-measures ANOVA revealed a significant effect of GNE-5729 (*F*_3,56_ = 7.55, *p* = 0.0003, ηp² = 0.29; Fig. 1D), while neither sex (*F*_1,23_ = 0.10, *p* = 0.75, ηp² = 0.004) nor the sex x treatment interaction (*F*_3,56_ = 0.41, *p* = 0.41, ηp² = 0.02) was significant. Post-hoc test showed that the 3 mg/kg dose significantly reduced arm entries compared to vehicle (*q* = 4.00, *p* = 0.0006), whereas the 0.75 and 1.5 mg/kg doses had no effect (*q* = 0.59, *p* = 0.89 and *q* = 0.25, *p* = 0.99, respectively). Separate analyses for females (Fig. 1E) and males (Fig. 1F) confirmed significant GNE-5729 effects on locomotor activity observed in both groups (*F*_3,27_ = 5.33, *p* = 0.005, ηp² = 0.37 and *F*_3,27_ = 4.02, *p* = 0.02, ηp² = 0.31 respectively; Supplementary Information, section 2.1.1).

Despite the absence of GNE-5729 dose effects in the multifactorial analysis, spontaneous alternations under vehicle conditions (vehicle injections) showed considerable variability. To assess whether GNE-5729 efficacy depended on baseline performance, we correlated the individual dose effect (GNE-5729 minus vehicle) with spontaneous alternations under vehicle conditions. This revealed a negative correlation between baseline performance and the effect of all three doses of GNE-5729. Animals with lower spontaneous alternations under vehicle conditions showed greater improvements following GNE-5729 treatment (Fig. 1G–I; 0.75 mg/kg GNE-5729: *r*^2^ = 0.42, *F*_1,22_ = 15.87, *p* = 0.0006; 1.5 mg/kg GNE-5729: *r*^2^ = 0.29, *F*_1,22_ = 8.86, *p* = 0.007; 3.0 mg/kg GNE-5729: *r*^2^ = 0.58, *F*_1,10_ = 13.61, *p* = 0.004), with generally stronger correlations in females than in males (cf. Fig. 1G–I).

Taken together, GNE-5729 did not affect spontaneous alternations at the group level; however, it improved performance in males when analyzed separately. Notably, GNE-5729 was most effective in mice with poor baseline performance under vehicle conditions, and it reduced locomotor activity at the highest dose tested (3 mg/kg).

#### GNE-5729 rescues pharmacologically impaired working memory

Based on the baseline-dependent effects, we next tested whether GNE-5729 could reverse working-memory deficits induced by dl-amphetamine, MK-801, or scopolamine.

##### GNE-5729 attenuates dl-amphetamine-induced deficits and hyperactivity

**Fig. 2:**
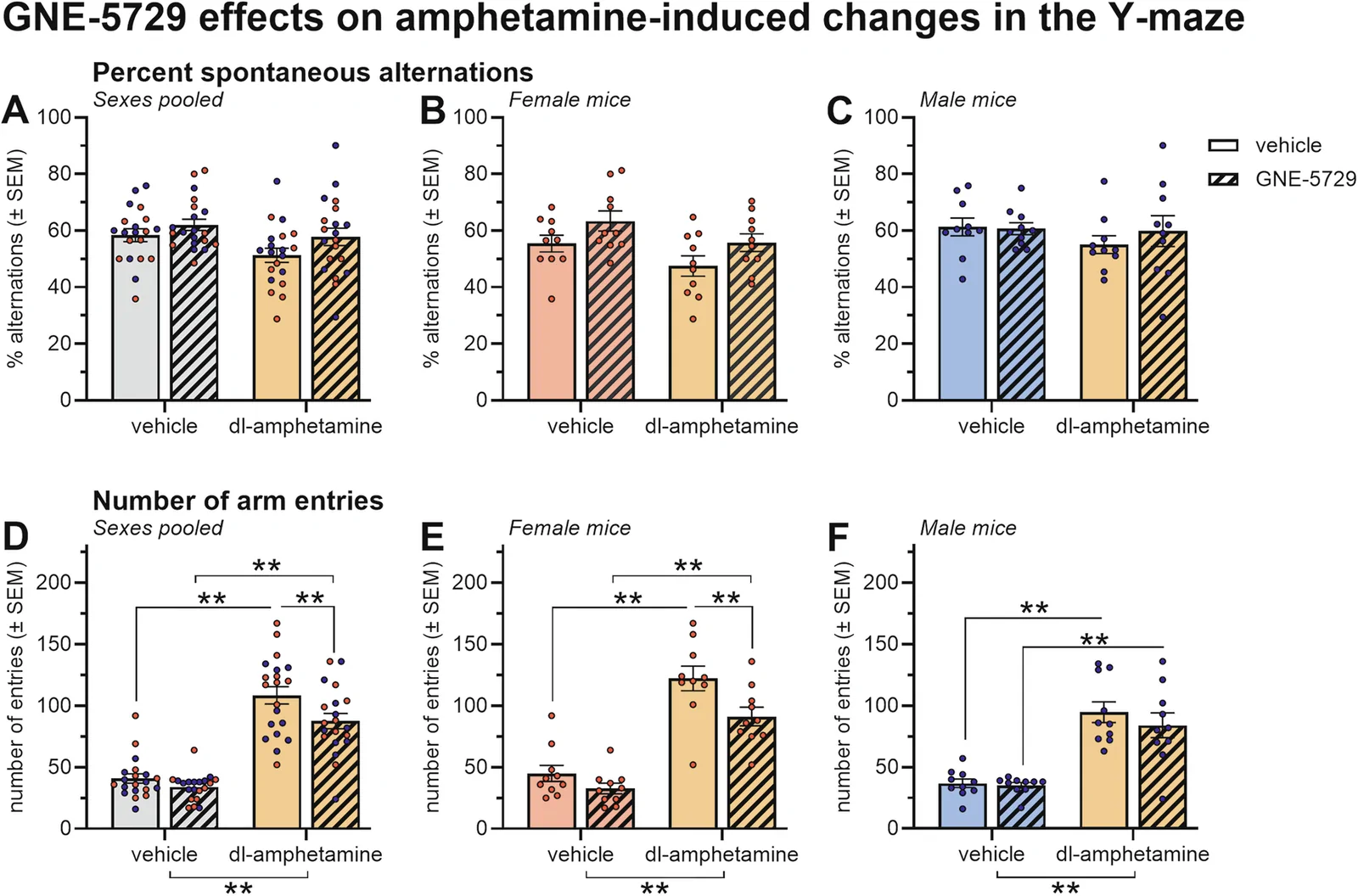
Effects of GNE-5729 on dl-amphetamine-induced impairments in working memory and locomotor activity. **A**–**C** Percentage of spontaneous alternations following vehicle or dl-amphetamine treatment with and without GNE-5729 co-administration. When sexes were pooled (**A**), dl-amphetamine reduced spontaneous alternations, and GNE-5729 significantly improved performance in dl-amphetamine-treated mice. Sex-separated analyses indicated clearer GNE-5729 rescue effects in females (**B**) than in males (**C**). **D**–**F** Number of arm entries as a measure of locomotor activity. dl-Amphetamine markedly increased locomotion, and GNE-5729 significantly attenuated this hyperactivity (**D**). Sex-separated analyses show that these effects were more pronounced in females (**E**) than in males (**F**). * *p* < 0.05, ** *p* < 0.01; post-hoc comparisons following significant ANOVA effects. Data represent mean ± SEM, with individual data points overlaid (red = female mice, blue = male mice).

A multifactorial repeated-measures ANOVA revealed a significant main effect of GNE-5729 (Fig. 2A; *F*_1,18_ = 5.60, *p* = 0.03, ηp² = 0.24) and a trend for a main effect for dl-amphetamine (*F*_1,18_ = 3.39, *p* = 0.08, ηp² = 0.16), with no main effects of sex and interactions (all *F*s < 1.97, *p*s > 0.17). The main effect of GNE-5729 reflects an overall increase in spontaneous alternations, which was primarily observed in dl-amphetamine-treated mice. Sex-separated analyses did not reveal significant effects, but GNE-5729 showed a trend toward significance in females (Fig. 2B; *F*s < 4.96, *p*s > 0.05) but not in males (Fig. 2C; *F*s < 0.84, *p*s > 0.38). As above, correlation analysis showed that the individual GNE-5729 effect was associated with spontaneous alternations under vehicle conditions: the poorer the vehicle performance, the greater the improvement with GNE-5729 (*r*^2^ = 0.47, *F*_1,18_ = 16.11, *p* = 0.0008). Of note, the rescue effect of GNE-5729 was not correlated with the deficit induced by dl-amphetamine (*r*^2^ = 0.07, *F*_1,18_ = 1.35, *p* = 0.26).

Analyses of the number of arm entries showed main effects of GNE-5729 treatment (Fig. 2D; *F*_1,18_ = 8.32, *p* = 0.01, ηp² = 0.32) and dl-amphetamine treatment (*F*_1,18_ = 206.70, *p* < 0.0001, ηp² = 0.92), with no sex effects (*F*_1,18_ = 2.42, *p* = 0.14, ηp² = 0.12) or interactions (*F*s < 3.01, *p*s > 0.10). dl-amphetamine robustly increased arm entries in both vehicle and GNE-5729-treated mice (*t*s > 8.81, *p*s < 0.0001), while GNE-5729 reduced the arm entries in dl-amphetamine-treated mice (*t* = 3.43, *p* = 0.003) but not in vehicle-treated mice (*t* = 1.13, *p* = 0.27). Effects were again stronger in females (Fig. 2E) than in males (Fig. 2F; Supplementary Information, section 2.1.2).

##### GNE-5729 does not rescue MK-801-induced deficits but attenuates hyperactivity in females

**Fig. 3:**
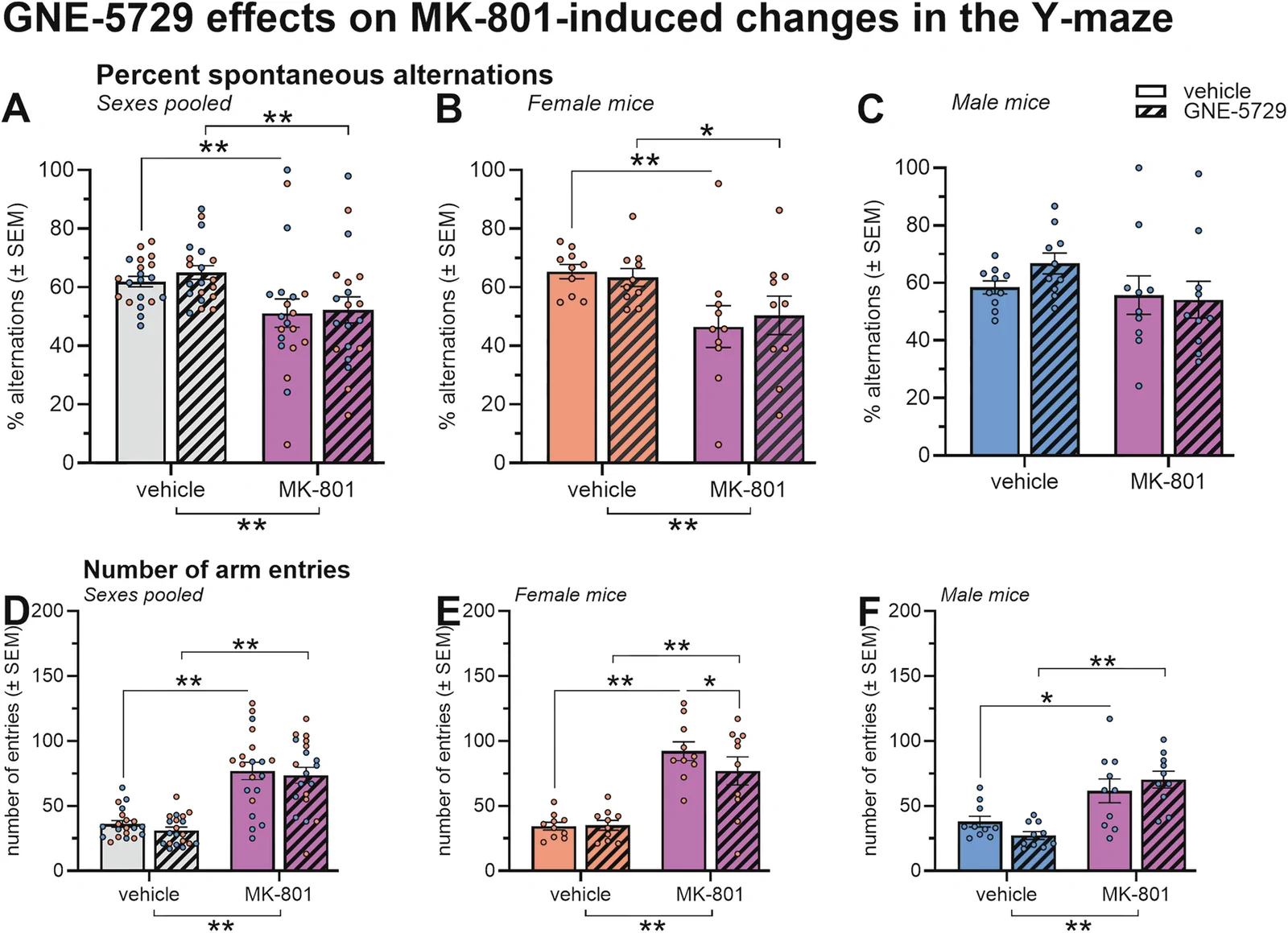
Effects of GNE-5729 on MK-801-induced impairments in working memory and locomotor activity. **A**–**C** Spontaneous alternations following vehicle or MK-801 administration with or without GNE-5729 co-treatment. MK-801 significantly reduced alternation performance in both treatment conditions (**A**), with more pronounced deficits observed in females (**B**) than in males (**C**). GNE-5729 did not rescue the MK-801-induced working memory impairment. **D**–**F** Number of arm entries as a measure of locomotor activity. MK-801 robustly increased locomotion (**D**), an effect observed in both sexes (**E, F**). GNE-5729 did not significantly alter this hyperactivity overall, although a partial attenuation of MK-801-induced increases in arm entries was found in females (**E**). * *p* < 0.05, ** *p* < 0.01; post-hoc comparisons following significant ANOVA effects. Data represent mean ± SEM. Individual data points are shown for each mouse (red = female mice, blue = male mice).

Analysis of spontaneous alternations using a multifactorial repeated-measures ANOVA revealed a significant main effect of MK-801 (Fig. 3A; *F*_1,18_ = 8.15, *p* = 0.01, ηp² = 0.31) and a trend toward an interaction between sex, GNE-5729 and MK-801 (*F*_1,18_ = 3.01, *p* = 0.099, ηp² = 0.14). All other main factors and interactions were not significant (*F*s < 1.00, *p*s > 0.34). Post-hoc comparisons indicated that MK-801 significantly impaired spontaneous alternations in both vehicle (*t* = 3.19, *p* = 0.005) and GNE-5729-treated mice (*t* = 3.80, *p* = 0.001). The effect was more pronounced in females (Fig. 3B) than in males (Fig. 3C). Consistent with previous findings, correlation analysis revealed a significant association between the individual GNE-5729 effect and spontaneous alternations under vehicle conditions (*r*^2^ = 0.31, *F*_1,18_ = 8.06, *p* = 0.01). In addition, the rescue effect of GNE-5729 was significantly associated with the deficit induced by MK-801 (*r*^2^ = 0.27, *F*_1,18_ = 6.53, *p* = 0.02), such that greater MK-801-induced impairments were associated with stronger GNE-5729-mediated improvements in performance.

A main effect of MK-801 on arm entries was observed (Fig. 3D; *F*_1,18_ = 87.60, *p* < 0.0001, ηp² = 0.83), with a significant sex×GNE-5729×MK-801 interaction (*F*_1,18_ = 5.83, *p* = 0.03, ηp² = 0.25), and trends for a GNE-5729 main effect (*F*_1,18_ = 3.48, *p* = 0.08, ηp² = 0.16) and sex×MK-801 interaction (*F*_1,18_ = 3.43, *p* = 0.08, ηp² = 0.16). Post-hoc analyses showed that MK-801 significantly increased arm entries in both vehicle and GNE-5729-treated mice (*t* = 7.03, *p* < 0.0001 and *t* = 7.32, *p* < 0.0001, respectively). These effects were confirmed in sex-separated analyses (Fig. 3E, F; Supplementary Information, section 2.1.3). Notably, in females, GNE-5729 significantly attenuated the MK-801-induced increase in arm entries (Fig. 3E; *t* = 2.49, *p* = 0.03).

##### GNE-5729 reverses scopolamine-induced deficits but not increased locomotion

**Fig. 4:**
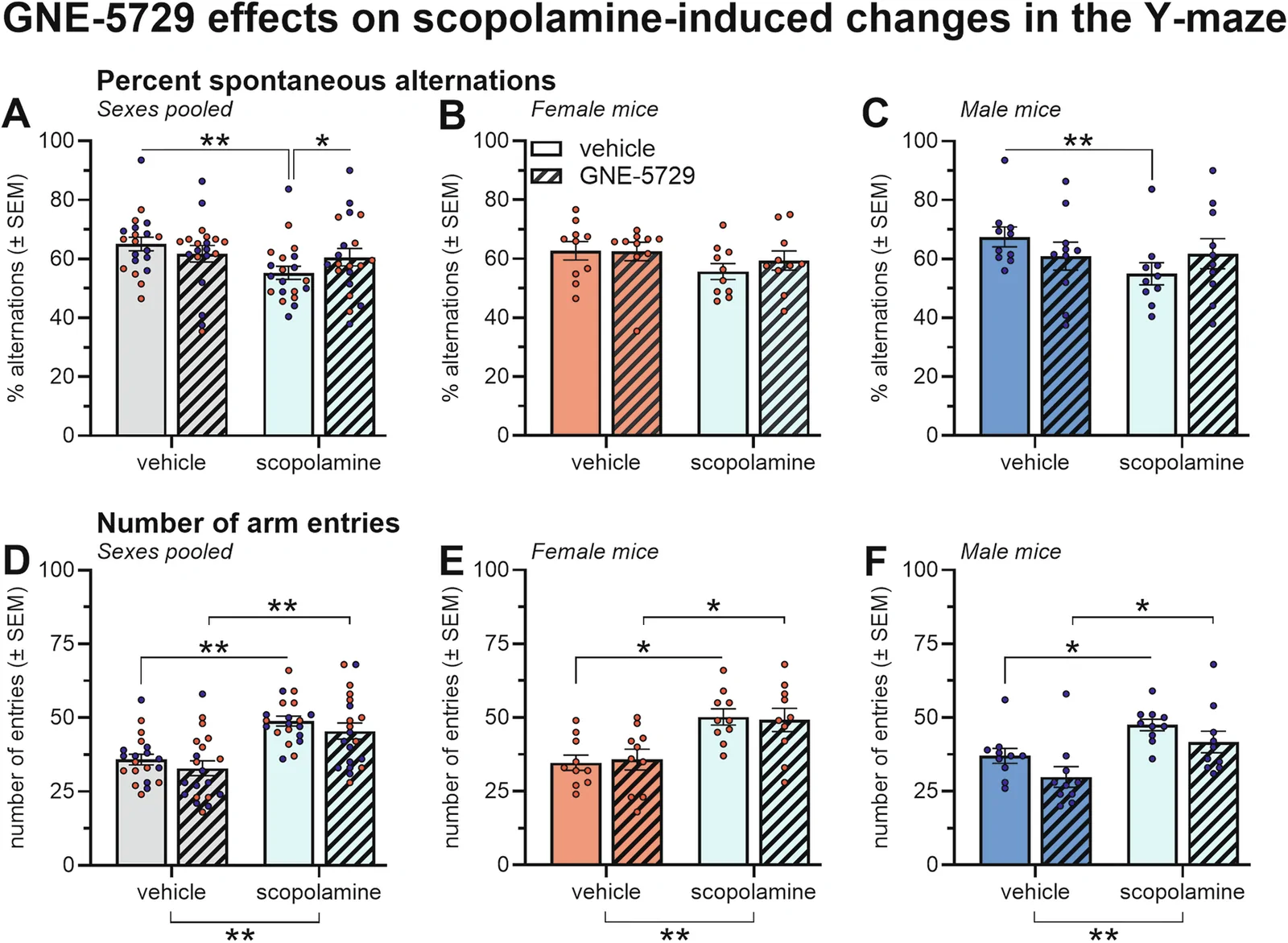
Effects of GNE-5729 on scopolamine-induced impairments in working memory and locomotor activity. **A**–**C** Spontaneous alternations in the Y-maze following vehicle or scopolamine administration with or without GNE-5729 co-treatment. Scopolamine reduced spontaneous alternations in vehicle-treated mice. GNE-5729 treatment increased spontaneous alternations in scopolamine-treated mice (**A**). Sex-separated analyses showed that GNE-5729 prevented scopolamine-induced impairments in males (**C**), whereas females did not show an effect of GNE-5729 (**B**). **D**–**F** Number of arm entries as a measure of locomotor activity. Scopolamine robustly increased locomotion in both treatment conditions (**D**), with similar effects observed in females (**E**) and males (**F**). GNE-5729 did not alter the scopolamine-induced increase in locomotor activity. * *p* < 0.05, ** *p* < 0.01; post-hoc comparisons following significant ANOVA effects. Data represent mean ± SEM, with data points representing individual mice (red = female mice, blue = male mice).

A multifactorial repeated-measures ANOVA revealed a significant GNE-5729×scopolamine interaction on spontaneous alternations (Fig. 4A; *F*_1,18_ = 5.72, *p* = 0.03, ηp² = 0.24) and a trend for a scopolamine main effect (*F*_1,18_ = 4.14, *p* = 0.06, ηp² = 0.19). All other main factors and interactions were not significant (*F*s < 1.22, *p*s > 0.28). Post-hoc comparisons revealed a significant effect reduction of spontaneous alternations by scopolamine in vehicle-treated (*t* = 3.87, *p* = 0.001) but not in GNE-5729-treated mice (*t* = 0.47, *p* = 0.64). Furthermore, GNE-5729 increased spontaneous alternations in scopolamine-treated mice (*t* = 2.07, *p* = 0.05). Sex-separated analyses showed that the interaction between GNE-5729 and scopolamine was not observed in females but was observed in males (Fig. 4B, C; *F*_1,9_ = 0.79, *p* = 0.40, ηp² = 0.08 and *F*_1,9_ = 5.90, *p* = 0.04, ηp² = 0.40, respectively). In line with this, scopolamine-induced impairments were observed in vehicle but not GNE-5729-treated males (Fig. 4C; *t* = 3.23, *p* = 0.01 and *t* = 0.20, *p* = 0.84, respectively). In contrast to the other experiments, the individual GNE-5729 effect was not significantly associated with spontaneous alternations under vehicle conditions (*r*^2^ = 0.15, *F*_1,18_ = 3.30, *p* = 0.09). Likewise, the rescue effect of GNE-5729 was not correlated with scopolamine-induced impairments (*r*^2^ = 0.0006, *F*_1,18_ = 0.01, *p* = 0.92).

Regarding the number of arm entries, there was a main effect of scopolamine (Fig. 4D; *F*_1,18_ = 35.71, *p* < 0.0001, ηp² = 0.67), while all other main effects and all interactions were non-significant (*F*s < 2.55, *p*s > 0.12). Post-hoc test showed that scopolamine increased arm entries in both vehicle and GNE5729-treated mice (*t* = 3.95, *p* = 0.0009 and *t* = 3.83, *p* = 0.001, respectively). These findings were confirmed in sex-separated analyses (Fig. 4E, F; Supplementary Information, section 2.1.4).

### GNE-5729 effects on cognitive flexibility

**Fig. 5:**
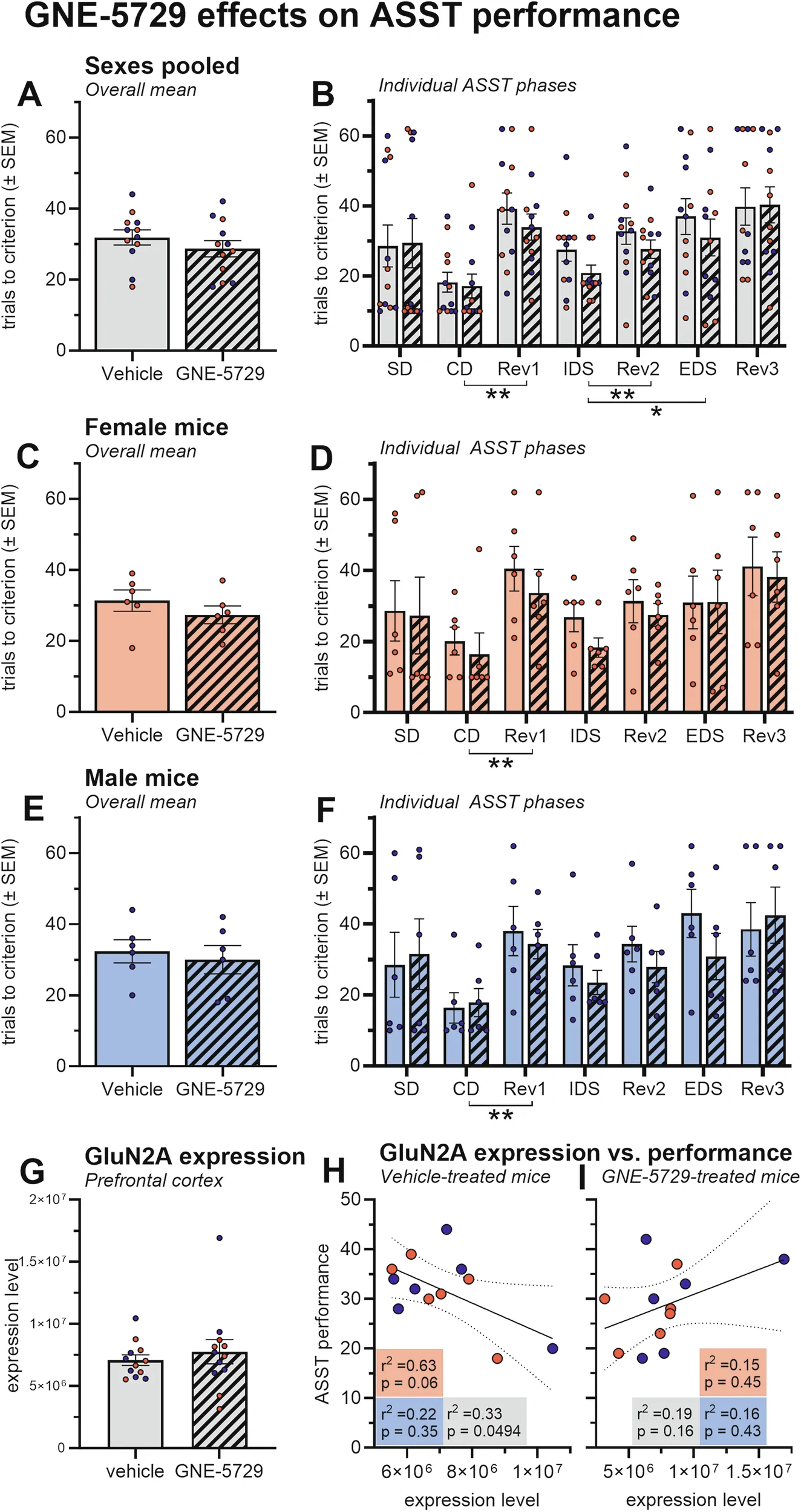
Effects of GNE-5729 on cognitive flexibility and prefrontal GluN2A expression. **A** Total trials to criterion across all ASST phases for vehicle and GNE-5729-treated mice. GNE-5729 did not significantly affect overall task performance. **B** Performance across the seven ASST phases, with more trials required in the reversal (Rev1, Rev2) and extradimensional shift (EDS) phases. **C**–**F** Sex-separated analyses for females (**C, D**) and males (**E, F**) showed no significant effects of GNE-5729 on performance. Trials to criterion varied across ASST phases in both females and males, with significant increases in trial number at Rev1 compared to the CD phase (**D, F**). **G** GNE-5729 did not alter GluN2A expression in the prefrontal cortex following four days of treatment. **H**–**I** Correlation between GluN2A expression and overall ASST performance. In vehicle-treated mice (**H**), GluN2A expression was significantly correlated with ASST performance, whereas no significant association was observed in GNE-5729-treated mice (**I**). * *p* < 0.05, ** *p* < 0.01; post-hoc comparisons following significant ANOVA effects. Data represent mean ± SEM (**A–G**), with individual data points overlaid (red female mice, blue male mice). Correlation statistics (r², p) for combined (gray), female (red), and male (blue) datasets are shown in the adjacent statistics boxes.

Cognitive flexibility was assessed using ASST performance, measured by the number of trials required to reach criterion (six consecutive correct choices) across phases. A multifactorial repeated-measures ANOVA showed no main effect of GNE-5729 (Fig. 5A; *F*_1,20_ = 1.05, *p* = 0.32, ηp² = 0.05), whereas ASST phase had a significant effect on performance (Fig. 5B; *F*_6,120_ = 5.89, *p* < 0.0001, ηp² = 0.23). Neither sex nor interactions were significant (*F*s < 0.27, *p*s > 0.61). Post-hoc comparisons revealed that more trials were required in the reversal phases Rev1 and Rev2 than in the preceding phases (*t* = 9.43, *p* < 0.0001 and *t* = 3.34, *p* = 0.003, respectively), and that EDS was more difficult than IDS (*t* = 2.22, *p* = 0.04).

Sex-separated analyses (Fig. 5C–F) confirmed that GNE-5729 had no significant main effects or interactions in either sex (*F*s < 1.02, *p*s > 0.33). However, a main effect of the ASST phase was observed in both groups, being less pronounced in females (Fig. 5D; *F*_6,60_ = 2.50, *p* = 0.03, ηp² = 0.20) than in males (Fig. 5F; *F*_6,60_ = 3.73, *p* = 0.003, ηp² = 0.27). Finally, similar to experiment 1, we examined whether the difficulty of an ASST phase under vehicle conditions was related to the GNE-5729-induced performance change (i.e., the difference in performance between GNE-5729 and vehicle treatment). Although mice generally required fewer trials under GNE-5729 treatment across most phases, there was no significant correlation between performance under vehicle conditions and the GNE-5729-induced change, neither when sexes were pooled (*r*^2^ = 0.11, *p* = 0.24) nor when analyzed separately (females: *r*^2^ = 0.002, *p* = 0.91; males: *r*^2^ = 0.24, *p* = 0.26).

Following the ASST experiment, we examined ex vivo whether GNE-5729 treatment affected GluN2A expression in the prefrontal cortex and whether GluN2A expression levels correlated with ASST performance. Four days of GNE-5729 treatment during the ASST had no effect on GluN2A expression compared to vehicle treatment (Fig. 5G; Mann-Whitney test: *U* = 60, *p* = 0.51). However, linear regression analysis revealed a significant association between GluN2A expression and ASST performance in vehicle-treated mice (Fig. 5H; *r*^*2*^ = 0.33, *p* = 0.049), but not in GNE-5729-treated mice (Fig. 5I; *r*^*2*^ = 0.19, *p* = 0.16). Notably, this correlation in vehicle-treated mice was much stronger in females (*r*^*2*^ = 0.63, *p* = 0.06) than in males (*r*^*2*^ = 0.22, *p* = 0.35).

## Discussion

In the present study, we investigated the pharmacodynamic effects of the GluN2A-selective PAM GNE-5729 [23] on EF in mice. GNE-5729 selectively improved working memory under suboptimal or neuromodulator‑disrupted states but had no effect on cognitive flexibility. Notably, its efficacy showed sex-dependent differences, with generally more pronounced effects in females.

GNE-5729 binds to the GluN1/GluN2A ligand-binding domain interface and potentiates NMDAR signaling only in the presence of endogenous co-agonists, reflecting a gating-dependent mechanism that selectively amplifies active glutamatergic transmission [6, 34]. This activity-dependence, consistent with other GluN2A selective PAM [35, 36], provides a mechanistic basis for state- or baseline-dependent effects. In line with this, our Y-maze findings showed that GNE-5729 modestly improved spontaneous alternations in males at 1.5 mg/kg and showed robust baseline performance-dependent efficacy across doses, particularly in females (Fig. 1). This pattern was replicated in our experiments employing pharmacologically induced working memory deficits. Notably, GNE-5729 effects were generally more closely associated with baseline working memory performance than with the magnitude of the pharmacologically induced impairment. Together, these findings suggest that GNE-5729 efficacy is shaped more strongly by baseline performance and the nature of the underlying deficit than by impairment severity alone. This aligns with the canonical inverted‑U models of neurotransmitter modulation of cognition, in which moderate NMDAR function shifts suboptimal subjects toward optimal performance, whereas those near peak performance drive limited benefit [37, 38]. Accordingly, mice with lower baseline performance gained the greatest cognitive benefit with GNE-5729, a pattern that parallels baseline-dependent pro-cognitive effects observed in humans with ADHD treated with methylphenidate [39–41] and similarly with other neuromodulators such as amphetamine [42] or memantine [43] in rodents. The absence of improvement in high-performing animals supports a compensatory enhancement model, in which GNE‑5729 potentiates GluN2A‑mediated signaling in hypofunctional networks, similar to activity-dependent effects reported for GNE‑0723, a close analog of GNE-5729 [36] but fails to elevate performance near ceiling levels, yielding a state-dependent rescue rather than a uniform cognitive enhancement.

At the highest dose tested (3 mg/kg), GNE-5729 markedly reduced locomotor activity, consistent with prior reports [44] indicating potential off-target effects rather than direct NMDAR modulation. Notably, the negative correlation between baseline performance and GNE-5729 efficacy was most pronounced at this dose. Because an NMDAR-mediated sedative effect would be expected to preserve or even enhance alternation performance, the dissociation between reduced locomotion and unchanged working memory suggests off-target interactions or excessive network dampening when NMDAR gain exceeds physiological limits. This indicates that GNE-5729 exhibits a narrow dose-response profile, with intermediate, non-sedating doses enhancing working memory in suboptimal states, whereas supra-optimal doses induce behavioral suppression that can confound locomotion-dependent cognitive measures.

The observed baseline-dependent effects motivated us to further characterize the state-dependent efficacy of GNE-5729. We tested its ability to rescue working memory deficits induced by three pharmacological challenges: dl‑amphetamine, scopolamine and MK‑801. Dl‑amphetamine, which disproportionately elevates synaptic dopamine and noradrenaline [45, 46] reduced spontaneous alternations and produced robust hyperactivity (Fig. 2), consistent with excessive dopaminergic stimulation impairing prefrontal-dependent cognitive processes [47] and destabilizing temporal coordination within prefrontal ensembles [48]. Co-administration of GNE-5729 improved alternation performance and attenuated hyperactivity, with effects primarily observed in dl-amphetamine-treated mice and a trend toward stronger effects in females. These findings suggest that GNE‑5729 may modulate circuit balance after monoaminergic challenge by state‑dependent potentiation of GluN2A‑containing NMDAR, as already shown for GNE‑0723 [25] to rebalance excitatory-inhibitory dynamics and suppress hypersynchrony.

Similarly, scopolamine impaired spontaneous alternations and increased locomotion (Fig. 4), consistent with its well-established profile as a muscarinic acetylcholine receptor antagonist producing amnestic and locomotor activity-enhancing effects [49–51]. Co-treatment with GNE-5729 reversed the alternation deficit without affecting the scopolamine-driven increase in locomotor activity, indicating selective rescue of memory. Similar selective cognitive rescues have been reported for D-cycloserine (DCS), a partial agonist at the NMDAR glycine binding site [52] which reverses scopolamine‑induced recognition deficits without changing total locomotor distance or exploratory metrics [53] and set-shifting impairments in ASST [27]. Because muscarinic receptor blockade disrupts cholinergic facilitation of NMDAR-dependent processes [54] GluN2A-selective NMDAR potentiation [25] may partially restore task performance, although intact cholinergic signaling is likely required for full recovery.

In contrast, the NMDAR antagonist MK-801-induced profound impairments in alternation and robust hyperactivity (Fig. 3), modeling schizophrenia-like cognitive impairment and stereotyped hyperactivity [55, 56]. GNE-5729 failed to rescue these deficits. Unlike dl-amphetamine or scopolamine, which perturb neuromodulatory regulation of intact glutamatergic circuits [57, 58]. MK-801 binds within the NMDAR channel pore [59] occluding receptor populations available for allosteric modulation. Although DCS and GluN2C/D-subunit PAM CIQ [60] can reverse MK‑801‑induced deficits [61, 62], they likely act through mechanisms compatible with suppressed channel activation. MK-801 also preferentially inhibits NMDAR on GABAergic interneurons, disinhibiting pyramidal cells and driving cortical hyperexcitation [63, 64], further limiting GluN2A PAM efficacy. Collectively, the state‑dependent, compensatory profile of GNE‑5729 appears constrained by the nature of the underlying deficit. These findings delineate mechanistic boundaries for GNE‑5729 efficacy, showing that it can improve performance following monoaminergic or muscarinic perturbations that leave NMDAR accessible but cannot overcome direct channel blockade, reflecting its dependence on intact, functional receptors for allosteric modulation.

Unlike its effects on working memory, GNE-5729 did not significantly affect cognitive flexibility in the ASST, suggesting differential effects across executive domains under the tested conditions. The ASST assesses the ability of mice to adapt their behavior in response to changing stimulus-reward contingencies [24, 65, 66]. As expected, we observed phase-dependent differences in performance, with more trials required for reversal and EDS phases [12, 22, 67]. However, GNE-5729 produced no effects across phases or sexes, and no treatment interactions emerged, demonstrating that acute GluN2A-selective PAM treatment does not enhance cognitive flexibility in otherwise healthy mice. Moreover, no baseline-response relationship was observed for ASST performance, as opposed to the Y-maze (Experiment 1), suggesting fundamental pharmacodynamic differences across executive domains. This also provides a principled rationale for not conducting pharmacological impairment experiments with the ASST.

Importantly, the absence of effects on cognitive flexibility does not necessarily contradict evidence from GluN2A loss-of-function models, as positive allosteric modulation may preferentially enhance performance under conditions of suboptimal or disrupted NMDAR signaling rather than in intact systems. Thus, the lack of effect in otherwise healthy mice may reflect limited capacity for further enhancement under near-optimal baseline conditions. In addition, ASST performance relies on dissociable prefrontal-orbitofrontal-striatal circuits [68–70] which may be less sensitive to GluN2A-mediated modulation than the medial prefrontal circuits engaged during working memory tasks. Systemic GluN2A modulation may also fail to selectively engage the circuits critical for phase-specific adaptations required by the ASST. Furthermore, healthy mice performing near ceiling on ASST may limit the scope for a GluN2A PAM to improve performance. Moreover, broad GluN2A modulation could simultaneously strengthen competing processes (e.g., goal-directed and habitual responses), resulting in offsetting effects that yield no net improvement on a complex, multi-component task, such as the ASST. These considerations suggest that GNE-5729’s modulatory effects may be restricted to working memory-related dynamics rather than the set-shifting mechanisms engaged by the ASST, reinforcing that set-shifting and working memory tap partly distinct mechanisms and may show different pharmacological sensitivity.

We also found that repeated GNE-5729 treatment over four days did not alter prefrontal GluN2A expression levels. However, GluN2A expression in vehicle-treated animals correlated with ASST performance (stronger in females), while this association attenuated in GNE‑5729-treated mice (Fig. 5). This suggests that endogenous GluN2A availability contributes to inter-individual differences in cognitive flexibility, as shown before by our group [12] and consistent with the finding that graded GluN2A depletion produces heterogeneous behavioral phenotypes [71]. The attenuation of this association following GNE‑5729 treatment may reflect reduced reliance of behavioral performance on endogenous GluN2A availability. Positive allosteric modulation may compensate for baseline differences in receptor expression, thereby reducing the influence of inter-individual variability on task performance. Alternatively, this pattern may indicate functional saturation of GluN2A-mediated signaling under GNE-5729 treatment.

It should be noted that a single dosing regimen of GNE-5729 was evaluated in the ASST. Although repeated administration was used, alternative dosing parameters (e.g., dose range, timing, or longer treatment duration) may be required to modulate cognitive flexibility. In addition, inclusion of a positive control compound known to improve ASST performance under impaired conditions (e.g., DCS [27] would further strengthen task sensitivity and should be considered in future studies.

Of note, females displayed greater sensitivity to GNE‑5729 across measures, a pattern that likely reflects sex‑dependent variation in GluN2A availability and NMDAR subunit composition. Estrous cycle stage was not monitored, which may have contributed to variability in female responses and should be considered when interpreting the observed sex differences. Estrous cycle-related fluctuations in cortical GluN2A [72] could create dynamic windows of receptor availability that increase sensitivity to a GluN2A‑selective PAM, while sex‑specific differences in frontal cortex neurochemistry may modulate glutamatergic signaling [73]. In addition, developmental [12] and cell‑type differences [72] in GluN2A expression may produce enduring circuit differences that influence drug responsiveness. This suggests that hormonal or sex‑linked network factors may shift females in a modulatory regime in which GluN2A potentiation is more amenable to pharmacological amplification via PAM such as GNE‑5729.

### Conclusion

The pharmacodynamic profile of GNE‑5729 suggests that GluN2A‑selective PAM are most beneficial under conditions of reduced but preserved NMDAR signaling, supporting a state‑dependent nootropic action. Observed sex differences across behavioral paradigms highlight the modulatory role of sex hormones on NMDAR function and underscore the importance of considering sex as a biological variable in preclinical studies. Future work should evaluate GNE‑5729 in models of NMDAR/GluN2A hypofunction (genetic, pharmacological, aging), incorporating sex-specific dosing or timing (e.g., estrous cycle stage) and circuit-specific or chronic paradigms to fully characterize its potential for improving cognitive flexibility. Collectively, these findings indicate that positive allosteric modulation of GluN2A‑containing NMDAR can rescue working memory under suboptimal or impaired conditions but does not uniformly enhance all executive domains, revealing both the therapeutic potential and mechanistic limits of GluN2A‑selective modulation.

## Supplementary information

Supplementary Information

## Data Availability

https://doi.org/10.5281/zenodo.19630908
