# Supplementary material for: Differential and state-dependent effects of the GluN2A-selective positive allosteric modulator GNE-5729 on executive functions
Source: Neuropsychopharmacology. 2026 Jun 25;51(11):1980–90. doi: 10.1038/s41386-026-02476-1 (PMC13598106; doi:10.1038/s41386-026-02476-1)
Supplement: Supplementary file 1 [file 41386_2026_2476_MOESM1_ESM.docx]

1. **MATERIALS AND METHODS**
   1.1. **Attentional set shifting task (ASST)**

During ASST, mice learned to associate specific stimuli with food rewards while ignoring irrelevant cues across multiple days [1, 2].

1.1.1. *Setup and materials*

The task was performed in a custom-built apparatus (41 cm × 20 cm × 20 cm) divided into three compartments: a waiting compartment and two choice compartments. A transparent wall separated the choice compartments, and two manually-operated transparent sliding doors connected them to the waiting compartment. Each compartment contained a bowl (5 cm diameter × 2.5 cm height). The bowl in the waiting compartment was filled with water, while the bowls in the choice compartments contained discriminative stimuli, one of which included a food reward (half of a chocolate rice; Choco-Krispies, Kellogs).

Two stimulus dimensions were used: visual/tactile and olfactory. Visual/tactile cues consisted of six filling materials: large yellow beads (M1), small yellow beads (M2), large plant clay beads (M3), small plant clay beads (M4), large aluminium foil balls (M5), and small aluminium foil balls (M6). Olfactory cues were applied to filter paper strips affixed to the outside of the bowls. At the start of testing, 30 µl of odorant (1:20 in paraffin oil) was applied, with an additional 10 µl added every hour. Odors included valeric acid (O1), S-(+)-carvone (O2), eucalyptol (O3), 2-phenylethanol (O4), citral (O5), and R-(-)-carvone (O6).

1.1.2. *Handling, food restriction, habituation, and pre-training*

Mice underwent a three-day handling phase, followed by food restriction to reduce bodyweight to 90-95% of free-feeding levels. On day 4, they were habituated to the bowls and the food reward by placing both in the home cage overnight.

On day 5, group habituation to the ASST apparatus took place. All animals from a cage were placed together in the ASST apparatus for approx. 30-45 min, with free access to all three compartments. Each choice compartment contained a bowl filled with home-cage bedding and baited with food rewards. This was followed by a 1-2 day rest period during which food restriction was maintained.

Individual pre-training was then conducted in the ASST apparatus. During this phase, both bowls were filled with home cage bedding, but only one contained the food reward. To prevent mice from locating the reward by odor, a small amount of powdered reward was sprinkled at the bottom of both bowls. The rewarded side (left or right choice compartment) was pseudorandomized. Each mouse completed 12 training trials. Initially, rewards were placed on top of the bedding; after every four trials, the reward was buried progressively deeper until, by the 9th trial, it was fully at the bottom of the bowl. If necessary, additional trials were conducted until the mice reliably dug for the reward.

1.1.3. *Test days*

The ASST consisted of seven phases conducted over four consecutive days. At the start of each phase, mice were placed in the waiting compartment. At trial onset, the sliding doors to the choice compartments were opened in a pseudorandomized sequence: either the rewarded/non-rewarded side first (with the second door opening upon entry), or both simultaneously. Mice then choose between the bowls located in the two choice compartments.

In the simple discrimination (SD), compound discrimination (CD), and intradimensional shift (IDS) phases (see below), each session began with four free trials during which mice could explore both bowls and correct their initial choice. In all subsequent trials and in all the remaining phases, a choice was defined as the mouse digging in one of the bowls. Once a choice was made, the door to the opposite choice compartment was closed to prevent correction. As in pre-training, the rewarded side followed a pseudorandomized order.

A phase was considered successfully completed when the mouse made six consecutive correct choices. If this criterion was not reached within 62 trials, the phase was classified as unlearned. In such cases, all 62 trials were included in the analysis, and the mouse proceeded to the next phase.

Day 1: simple discrimination (SD)

Cue exemplars from a single stimulus dimension were presented. Mice were required to associate one exemplar with the food reward. For example, when visual/tactile cues were used, M1 was rewarded and M2 was not.

Day 2: compound discrimination (CD) and compound discrimination reversal (Rev1)

A second, task-irrelevant stimulus dimension (e.g., O1 and O2) was introduced. Reward remained contingent on the original dimension, independent of the added stimuli. Thus, M1/O1 and M1/O2 were rewarded, whereas M2/O1 and M2/O2 were not.

Upon successful completion of CD, mice immediately proceeded to Rev1, in which the reward contingency within the relevant dimension was reversed. In this phase, M2/O1 and M2/O2 were rewarded, while M1/O1 and M1/O2 were not. CD and Rev1 were completed sequentially on the same day.

Day 3: intradimensional shift (IDS) and intradimensional shift reversal (Rev2)

All cue exemplars from both stimulus dimensions were replaced with novel stimuli, but the relevant dimension remained unchanged. For example, in IDS, M3/O3 and M3/O4 were rewarded, whereas M4/O3 and M4/O4 were not.

In Rev2, the reward contingency within the relevant dimension was reversed: M4/O3 and M4/O4 were rewarded, while M3/O3 and M3/O4 were not.

Day 4: extradimensional shift (EDS) and extradimensional shift reversal (Rev3)

As in the IDS, novel exemplars from both dimensions were introduced. However, in contrast to previous phases, the previously irrelevant dimension now became relevant. For example, reward was associated with O5/M5 and O5/M6, whereas O6/M5 and O6/M6 were not.

In Rev3, the contingency within the newly relevant dimension was reversed, such that O6/M5 and O6/M6 were rewarded, while O5/M5 and O5/M6 were not.

1.2. **Molecular analyses**
     1.2.1. ***Brain dissection and tissue collection***

After completing the final phase of ASST (Rev3), mice were anesthetized with isoflurane (CP-Pharma # 1214; Burgdorf, Germany), decapitated and their whole brains were immediately removed. Brains were transferred to an ice-chilled mouse coronal brain slicer matrix (RBM-2000C, ASI-instrument, Warren, MI, USA) and cut into 1 mm coronal sections. The prefrontal cortex (PFC) was scalpel-dissected, weighed and stored at -80° C until further processing.

1.2.2. ***Crude synaptosomal membrane preparation***

Crude synaptosomal fractions were prepared from the PFC tissue as described previously [3], with minor modification. Briefly, samples were homogenized at 1000 rpm in ice-cold lysis buffer (0.32 M sucrose solution containing 5 mM HEPES pH 7.4, protease inhibitors (cOmplete™ Protease Inhibitor Cocktail, 04693132001, Roche; Merck, Darmstadt, Germany), and phosphatase inhibitors (PhosSTOP™, 04906837001, Roche; Merck, Darmstadt, Germany)) using KaVo K5 plus handpiece (type 4911, Kaltenbach & Voigt gmbh, Biberach/Riß, Germany) controlled via a connected foot control ‘KaVo K-Control TLC 4957’ unit (Kaltenbach & Voigt gmbh, Biberach/Riß, Germany). The homogenate was centrifuged at 1000 g for 5 min at 4º C to separate nuclei (pellet P1) from the supernatant (S1). S1 was centrifuged again at 12,000 g for 20 min at 4 °C, yielding supernatant S2 and pellet P2. Pellet P1 and supernatant S2 were collected and stored, while pellet P2 was resuspended in sodium dodecyl sulfate for subsequent analysis. Protein concentration was determined using the amidoblack protein assay [4].

1.2.3. ***Capillary-based Western immunoassay***

Protein expression was assessed using automated capillary-based Western immunoassay on the Jess system (004-650, ProteinSimple, Bio-Techne, Wiesbaden, Germany), which employs capillary electrophoresis for protein separation and immunoassay-based chemiluminescent detection [2, 3]. The assay was performed under standard conditions recommended by the manufacturer, using the 12-230 kDa Separation Module (SM-W004, ProteinSimple, Bio-Techne, Wiesbaden, Germany) and the anti-rabbit detection module (DM-001, ProteinSimple, Bio-Techne, Wiesbaden, Germany).

Samples were diluted to 0.4 mg/ml with 0.1x sample buffer (diluted from 10x buffer, provided in SM-W004) and mixed at a 1:4 ratio with the 5x fluorescent master mix (EZ standard pack, SM-W004). The mixture was denatured at 95°C for 5 min, vortexed, centrifuged, and stored on ice. Samples, primary anti-NMDAR2A antibody (1:10, AB1555P, Millipore, Darmstadt, Germany), secondary anti-rabbit antibody and the remaining detection reagents (DM-001) were then loaded into the 12-230 kDa pre-filled assay plate (SM-W004) per manufacturer instructions.

Total protein quantification was performed using the Total Protein Detection Module for chemiluminescence (DM-TP01, ProteinSimple, Bio-Techne, Wiesbaden, Germany). The RePlex Module (RP-001, ProteinSimple, Bio-Techne, Wiesbaden, Germany) facilitated antibody removal following the initial detection, permitting subsequent total protein quantification within the same capillary. The prepared assay plate and capillary cartridge (SM-W004) were placed inside the Jess system for capillary electrophoresis and chemiluminescence-based detection. Data were visualized as electropherograms, and peak areas corresponding to target protein were quantified using Compass for SW software (version 6.3.0).

2. **RESULTS**
   2.1. **Sex-separated analyses of arm entries**
        2.1.1. *Dose-response effects of GNE-5729*

For the sex-separated analyses of arm entries, post-hoc comparisons showed that the 3 mg/kg dose significantly reduced arm entries compared with vehicle in both females (*q* = 3.07, *p* = 0.01) and males (*q* = 3.26, *p* = 0.009).

2.1.2. *Effects of GNE-5729 on* *dl-amphetamine-induced impairment*

In females, main effects of GNE-5729 treatment (F_1,9_ = 9.55, *p* = 0.01, ηp² = 0.52) and dl-amphetamine treatment (F_1,9_ = 193.7, *p* < 0.0001, ηp² = 0.96) were observed. Post-hoc comparisons confirmed dl-amphetamine effects in both vehicle (*t* = 9.32, *p* < 0.0001) and GNE-5729-treated mice (*t* = 7.04, *p* < 0.0001). GNE-5729 reduced arm entries in dl-amphetamine-treated mice (*t* = 2.25, *p* = 0.005). In males, only a main effect of dl-amphetamine treatment was observed (F_1,9_ = 60.1, *p* < 0.0001, ηp² = 0.87). Post-hoc comparisons confirmed dl-amphetamine effects in both vehicle (*t* = 6.29, *p* = 0.0001) and GNE-5729-treated mice (*t* = 5.30, *p* = 0.0005).

2.1.3. *Effects of GNE-5729 on* *MK-801-induced impairment*

A main effect of MK-801 treatment was observed in females (F_1,9_ = 53.59, *p* < 0.0001, ηp² = 0.86) and males (F_1,9_ = 34.07, *p* < 0.0001, ηp² = 0.79). Post-hoc comparisons confirmed MK-801 effects in both vehicle- and GNE-5729-treated females (*t* = 9.41, *p* < 0.0001 and *t* = 6.80, *p* < 0.0001, respectively) and males (*t* = 2.85, *p* = 0.02 and *t* = 5.17, *p* = 0.0006, respectively).

2.1.4. *Effects of GNE-5729 on* *scopolamine-induced impairment*

A main effect of scopolamine treatment was observed in females (F_1,9_ = 13.73, *p* = 0.005, ηp² = 0.60) and males (F_1,9_ = 39.46, *p* = 0.0001, ηp² = 0.81). Post-hoc comparisons confirmed significant scopolamine effects in both vehicle- and GNE-5729-treated females (*t* = 3.03, *p* = 0.01 and *t* = 2.62, *p* = 0.03, respectively) and males (*t* = 2.40, *p* = 0.04 and *t* = 2.70, *p* = 0.02, respectively).

**References**

1.	Duske J, D'Souza N, Mayer D, Dieterich DC, Fendt M. Orexinergic modulation of chronic jet lag-induced deficits in mouse cognitive flexibility. Neuropsychopharmacology. 2025;50(5):762-71. doi:10.1038/s41386-024-02017-8

2.	Afzal S, Durrast N, Hassan I, Soleimanpour E, Tsai PL, Dieterich DC, et al. Probing cognitive flexibility in Shank2-deficient mice: Effects of D-cycloserine and NMDAR signaling hub dynamics. Prog. Neuropsychopharmacol. Biol. Psychiatry. 2024;134:111051. doi:10.1016/j.pnpbp.2024.111051

3.	Seifried L, Soleimanpour E, Dieterich DC, Fendt M. Cognitive Flexibility in Mice: Effects of Puberty and Role of NMDA Receptor Subunits. Cells. 2023;12(9). doi:10.3390/cells12091212

4.	Badin J, Herve B. [Protein Precipitation by Amidoschwarz and Its Application to the Microdetermination of Blood Proteins]. Ann. Biol. Clin. (Paris). 1965;23:321-32.
